# High albedo daytime radiative cooling for enhanced bifacial PV performance

**DOI:** 10.1515/nanoph-2023-0611

**Published:** 2023-12-14

**Authors:** Hannah Kim, Yiwei Gao, Ethan Moran, Annyn Howle, Sean McSherry, Spencer Cira, Andrej Lenert

**Affiliations:** University of Michigan, Ann Arbor, MI, USA

**Keywords:** radiative cooling, ground albedo, bifacial, photovoltaics

## Abstract

We present a radiative cooling material capable of enhancing albedo while reducing ground surface temperatures beneath fielded bifacial solar panels. Electrospinning a layer of polyacrylonitrile nanofibers, or nanoPAN, onto a polymer-coated silver mirror yields a total solar reflectance of 99 %, an albedo of 0.96, and a thermal emittance of 0.80. The combination of high albedo and high emittance is enabled by wavelength-selective scattering induced by the hierarchical morphology of nanoPAN, which includes both thin fibers and bead-like structures. During outdoor testing, the material outperforms the radiative cooling power of a state-of-the-art control by ∼20 W/m^2^ and boosts the photocurrent produced by a commercial silicon cell by up to 6.4 mA/cm^2^ compared to sand. These experiments validate essential characteristics of a high-albedo radiative-cooling reflector with promising potential applications in thermal and light management of fielded bifacial panels.

## 1 Introduction

Regulating the temperature and diffuse light levels around fielded solar panels offers an opportunity to improve the performance and lifetime of existing and future photovoltaic (PV) installations. Solar heating significantly degrades both the efficiency (by ∼0.4 % °C^−1^) and lifespan (by ∼7 % °C^−1^) of solar panels [1]. Meanwhile, a large fraction of sunlight incident on the overall area of the solar installation remains unused. Even minor improvements in the performance of fielded panels can vastly impact the global energy system, given the expected multi-terawatt deployment of solar PV [2]. Tracking and bifacial PV technologies, which are being increasingly deployed in utility-scale solar installations, stand to particularly benefit from such thermal and light management.

Engineering the optical and thermal properties of the materials beneath and around the PV panels is gaining interest as a way to improve illumination and reduce panel temperatures. Research in agrivoltaics has shown that crops can reduce the local air temperature at the PV site, thereby reducing panel temperatures by up to 10 °C [3]. Meanwhile, simulations indicate that replacing natural groundcovers with artificial ground reflectors should substantially improve power output and energy yield of bifacial PVs [4–6]. Increasing ground albedo from 0.25 (typical for natural groundcover) to 0.50 can increase the bifacial electricity yields by 20 % (global average) [4]. Reaching the highest bifacial gains requires optimizing the elevation and orientation of bifacial panels depending on latitude and albedo. Nonetheless, the promise of improved yields, easier cleaning (in a vertical orientation), and compatibility with leading PV technologies has driven growing interest in bifacial PV. That growth also motivates efforts to increase ground albedo and shape the landscape of the solar field [7].

Regarding thermal management, radiative cooling has emerged as a promising means of passively regulating temperature, with various applications including energy savings in buildings [8], atmospheric dew harvesting [9, 10], and enhancing personal thermal comfort [11, 12]. Daytime radiative cooling has been achieved by combining very high solar reflection (>90 %) with good thermal emission, specifically within the atmospheric transparency windows (8–13 µm and 16–28 µm) [13, 14]. Given that peak solar irradiation (∼1000 W/m^2^) imparts about 10 times more heat than can be rejected by radiative cooling alone (∼100 W/m^2^), a 1 % increase in solar reflectance can produce a cooling power gain of approximately 10 W/m^2^ [15, 16]. This insight has made suppressing solar heating a top priority, with various approaches such as multilayer emitters [17–20], particle-based coatings [21–27], and transparent insulating covers [28– 34] used to achieve high solar-weighted reflectance (SR) [35, 36]. Nonetheless, few approaches attain very high radiative cooling while providing the high albedo desired for redirecting sunlight toward bifacial panels. Furthermore, glare is an issue with many existing radiative coolers which can hinder the adoption of solar technologies [37–39].

**Figure 1:**
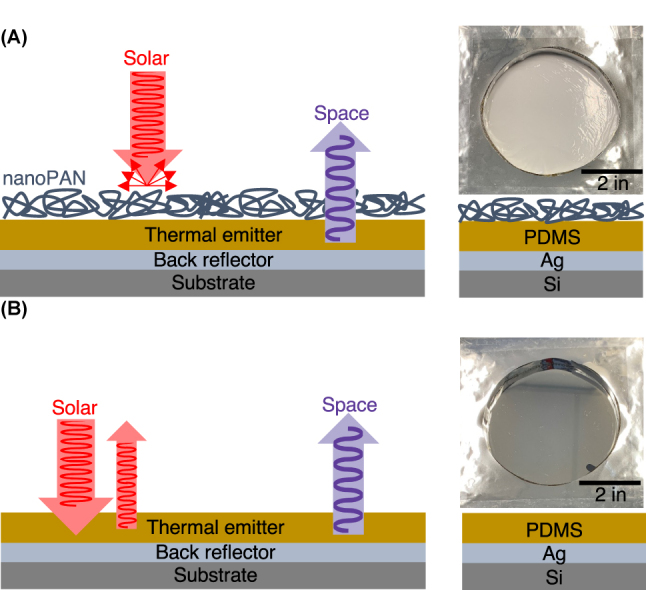
Layered structures and overhead images of the radiative-cooling reflectors: (a) the PAN nanofiber (nanoPAN) artifical ground reflector (nanoPAN/PDMS/Ag) with a high albedo. (b) the polymer-mirror control (PDMS/Ag) that represents a state-of-the-art specular radiative cooler.

Here, we fabricate and investigate materials with a combination of high albedo and excellent radiative cooling performance for potential use as an artificial ground reflector in bifacial solar installations. Figure 1 shows representative images of the structure fabricated by coating a silicon wafer with a thin layer of silver as the back reflector (mirror), polydimethylsiloxane (PDMS) as the thermal emitter layer, and eletrospun polyacrylonitrile nanofibers (nanoPAN) as the high aldebo layer. Though other methods such as phase separation [32], phase-inversion [28, 33], and sacrificial particle templating [31] can be used to fabricate porous polymer films, electrospinning provides excellent control over the morphology and polydispersity at length scales relevant to solar radiation [23, 29, 30]. The applied nanoPAN layer enhances reflectance in the ultra-violet (UV) and near infrared (IR) regions, resulting in a total solar-weighted reflectance of 99 ± 0.5  %. Radiative cooling tests under clear sky conditions in Ann Arbor, MI, show temperature reduction and cooling power enhancements of ∼3 °C and ∼20 W/m^2^, respectively, compared to the polymer-mirror control (without nanoPAN). The control is intentionally similar to an emitter that achieved state-of-the-art daytime radiative cooling performance under clear sky conditions in California [20], including cooling to 8 °C below ambient and a cooling power of ∼127 W/m^2^ at ambient. Furthermore, replacing sand with the nanoPAN-based reflector increases the photocurrent produced by a ground-facing latitude-tilted commercial silicon cell by as much as 6.42 mA/cm^2^. This result corresponds to an estimated 18 % enhancement relative to the output of a typical silicon cell. Overall, the results shown here demonstrate the effectiveness of nanoPAN in enhancing radiative cooling and backside illumination. It is important to note that these functions of the material are demonstrated separately and at the scale of an individual cell in this proof-of-concept study. To evaluate the effects of the artificial ground reflector on the working temperatures and energy yields of bifacial panels, future field testing in utility-scale installations is necessary. Specifically, additional field research is needed to assess the impact of ground-surface temperatures and albedo on the microclimate at the PV site and the panel temperatures. There is not enough information in literature to empirically estimate this effect, particularly across a broad range of climates and field types.

## 2 Materials and methods

### 2.1 Fabrication

The back reflector (10 nm Ti/150 nm Ag) is deposited by electron beam deposition onto a four inch Si wafer. The thermal emitter layer (150 μm polydimethylsiloxane, *Dow Sylgard 184*) is added by spinning coating and cured for 15 min at 150 °C. The nanoPAN layer is deposited as previously [30] using a home-built electrospinning setup. The fiber morphology is optimized by controlling spinning parameters and the quantity of PAN in solution. PAN powder (*Polysciences Inc.*) with an average MW of 200,000 is fully dissolved in dimethylformamide (*Sigma*) at 6 wt% concentration by mixing overnight at 40–50 °C. The solution is loaded into a syringe with a 25-gauge blunt tip needle and placed in a syringe pump to ensure a constant flow rate. The PAN solution is electrospun at a flow rate of 0.4 mL h^−1^ and stage height of 11.5 cm for 80 min.

### 2.2 Radiative cooling measurements

Outdoor radiative cooling performance is evaluated as previously [30] using a custom rooftop test station in Ann Arbor (MI, USA). A transparent polyethylene convective cover is used. The variation of the global horizontal irradiance, on-site ambient temperature (measured using a shaded temperature logger adjacent to the samples, see [30] for details), and sample temperature is monitored throughout the tests. The reported cooling power of the nanoPAN sample is determined using outdoor measurements of its temperature with and without a constant heat input. The heated setup is identical to the stagnation temperature measurement except that a resistive heater is adhered to the sample with nanoPAN. Constant power is supplied to the heater and the resulting temperature of the sample is measured. The measured power input and the temperatures of the resistively heated and unheated samples are used to calculate an effective heat transfer coefficient (HTC) between the sample and the ambient, which is in turn used to find the cooling power at ambient temperature throughout the day. The assumption of a constant HTC is justified by the narrow temperature range (less than 10 °C). Similarly, the ambient-temperature cooling power for the control is determined using its measured stagnation temperature and the effective HTC described above. Differences in HTC are neglected because of the similarity in insulation and thermal emittances of the sample and control.

### 2.3 Backside illumination tests

Commercial silicon photovoltaic cells (*SunPower, Me1*) are mounted on a sand covered surface with an albedo value of approximately 30 %, which is close to the Earth’s average albedo. The orientation of the photovoltaic cells is set to 42° tilt angle (local latitude of Ann Arbor, MI, USA). The distance between the bottom edge of the PV cell and the edge of the circular reflector is 5 cm. The open-circuit voltage, *V*_OC_, generated by the PV cells with and without a 10 cm-diameter circular nanoPAN-based reflector is measured by a multimeter. The photovoltaic cells used in the tests are monofacial with the front surface (blue) facing true north (ground). The ground-facing monofacial cell mimics the backside of a bifacial solar cell and serves to determine a representative measure of the added photocurrent resulting from the higher ground albedo, which is the primary goal of this experiment. The measured voltage is averaged over three 20-minute periods, corresponding to 3 hours before solar noon, solar noon, and 3 hours after solar noon.

Based on the experimentally measured *V*_OC_ values, the *I*_*L*_ is calculated using a single diode approximation given by:

(1) $$I_{\text{L}}=I_{0}\left(e^{\frac{qV_{\text{OC}}}{nkT}}-1\right)$$

where *I*_0_ is the dark saturation current, *n* is the ideality factor, *k* is the Boltzmann constant, *T* is temperature of PV cell, and *q* is electron charge. *I*_0_ and *n* are determined by fitting variable-illumination current-voltage curves using established procedures [40], which yields *n* = 1.4 and *I*_0_ = 1.118(10^−7^) A.

## 3 Results and discussions

### 3.1 Radiative properties of nanoPAN

**Figure 2:**
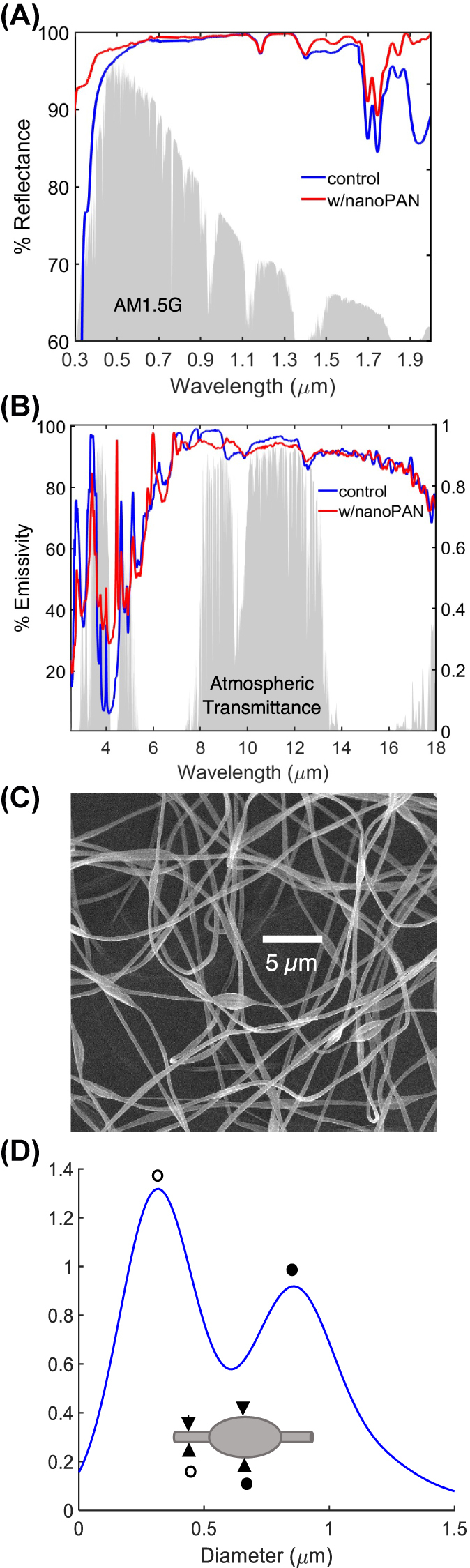
Radiative properties of the reflectors and morphology of the beaded nanoPAN fibers. (a) Spectral total reflectance measured by UV–Vis and (b) spectral emissivity measured by FTIR for samples with and without the nanoPAN layer. The AM1.5 G and representative atmospheric transmittance spectra are shown for reference [41]. (c) SEM image of the 6 wt% electrospun nanoPAN fibers deposited using electrospinning. (d) Hierarchical size distribution of the interconnected fiber (white) and bead-like (black marker) features.

The variation of the total reflectance *versus* wavelength for the nanoPAN-based reflector (nanoPAN/PDMS/Ag) and mirrored control (PDMS/Ag) are shown in Figure 2a and b. The results for the control show lower reflectance starting around 0.5 µm and an overall SR of 97 %. The addition of the nanoPAN layer increases the total solar reflectance to 99 ± 0.5 %. Substantial increases in reflectance are observed at wavelengths below 0.5 µm and between 1.4 and 2 µm. In contrast, the nanoPAN layer has a small effect on the long-wavelength properties relevant to thermal emission. The atmospheric weighted thermal emittance (*ɛ*) decreases only from 83 to 80 % with the nanoPAN.

The key to achieving the shown selective enhancement in solar reflectance is the well-controlled hierarchical morphology of the nanofibers, which features cylindrical and ellipsoidal (bead-like) geometries. Figure 2c and d show images of the fabricated nanoPAN films and the bimodal size distribution associated with the fibers and beads obtained from >10 different SEM images using image processing software. Due to their characteristic length scales, the fibers are responsible for scattering shorter wavelengths and the beads for scattering longer solar wavelengths, as supported by simulations [29, 30].

Overall, the addition of nanoPAN increases the total SR by 2 % by addressing spectral ranges where the control is less reflective. Though this improvement may seem small, a 2 % improvement in SR (and negligible change in thermal emittance) is expected to yield a ∼20 W/m^2^ gain in daytime cooling power. Furthermore, the nanoPAN layer increases the diffuse reflectance (or albedo) to 95.6 ± 0.5 % (Supplementary Material Figure S1), which is key to improving light collection in bifacial installations and gives the films a soft white appearance, minimizing unwanted glare. In addition, applying the nanoPAN layer to a PDMS-coated aluminum sheet increases solar reflectance from 77 to 94 % (Supplementary Material Figure S2), demonstrating that the approach can be translated to lower cost substrates.

### 3.2 Radiative cooling performance

**Figure 3:**
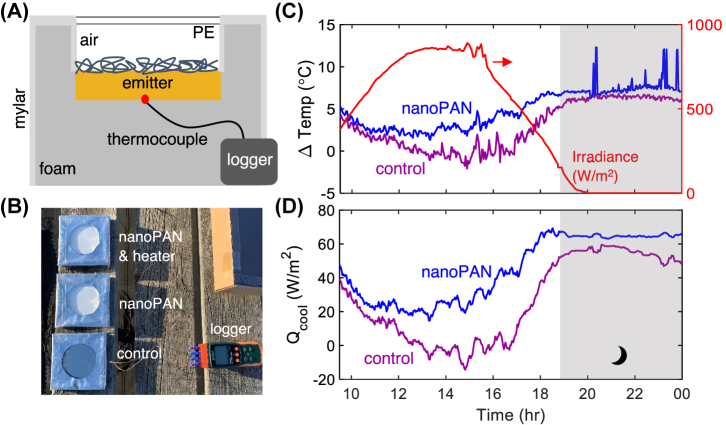
Increase in radiative cooling performance due to the nanoPAN layer. Schematic (a) and image (b) of the outdoor setup used to evaluate radiative cooling performance. (c) Temperature reduction (*i.e*., ambient temperature minus the sample stagnation temperature; positive indicates greater sub-cooling) comparing the nanoPAN sample to the control. (d) Corresponding cooling power at ambient temperature (a 10-min moving average is applied to decrease noise).

The radiative cooling performance of the nanoPAN sample (99 % SR) and its mirrored control (97 % SR) are assessed under clear sky conditions. Figure 3 shows the difference between the ambient temperature and the stagnation temperature (Δ*T* = *T*_amb_ − *T*_emit_), which is known as the temperature reduction or sub-ambient temperature. During the daytime, the temperature of the control hovers around ambient temperature while the nanoPAN sample is consistently below ambient. At night, both materials approach similar sub-ambient temperatures. The day to night difference in temperature reduction is approximately 9 °C for the control and 4 °C for the nanoPAN sample. These results show that temperature of the nanoPAN ground reflector is less sensitive to variations in solar irradiance. Similar improvements relative to the control were observed on other test days with small differences due to variable weather conditions.

In addition to the stagnation temperature, the ambient-temperature cooling power (Figure 3d) is relevant to thermal management in solar fields. The average cooling power of the nanoPAN sample over the 14-h period is ∼48.2 W/m^2^. Imperfect sky conditions and parasitic heating of the enclosure are likely responsible for a lower cooling power than previously reported for emitters with comparable radiative properties, including the polymer-mirror emitter mentioned above [20]. Importantly, the afternoon cooling power increases by ∼20 W/m^2^ due to the addition of the nanoPAN layer, consistent with the 2 % SR gain. One would expect that additional cooling power to translate to locations with utility-scale PV installations, most of which have better sky conditions than Ann Arbor, MI.

### 3.3 Backside illumination enhancement

**Figure 4:**
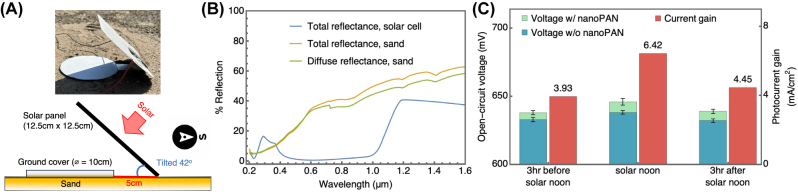
Enhanced backside PV illumination enabled by the high-albedo reflector. (a) Orientation and geometry of the outdoor backside illumination test. (b) Optical properties of the cells and the sand surface used in the experiments as measured by UV–Vis. (c) The nanoPAN reflector boosts the open-circuit voltage of the cells. Corresponding current gains, attributed to the improved light harvesting, are estimated using an empirical cell model.

In the backside PV illumination tests, we analyzed the current and voltage gains mediated by the addition of the nanoPAN ground reflector in a latitude-tilted north-south orientation, common in solar installations. The orientation and geometry of the field tests are shown in Figure 4a. Sand is used as the baseline surface for these comparative tests because its albedo, shown in Figure 4b, approximately matches the Earth’s average. In contrast, the albedo of the nanoPAN ground reflector is comparable to snow [42].

During the testing period, the nanoPAN-based reflector consistently enhances the *V*_OC_ generated by the commercial PV cell compared to the sand surface. The enhancement reaches its highest value at solar noon. The voltage gain is attributed to additional light collected by the cell. Using the empirical cell model described above, we estimate absolute current gains ranging from 3.93 to 6.42 mA/cm^2^ depending on the specific time of day, with the highest gain observed at solar noon. To put the current gains in context, a typical silicon solar cell produces around 35 to 40 mA/cm^2^ under standard test conditions. Thus, the nanoPAN cover can add a substantial amount of current, and therefore power, to the output of a cell, even after accounting for typical bifaciality factors of 0.74–0.95 [43–45]. Provided that the relative geometry between the reflector and the backside of the cells is unchanged, the backside illumination enhancements shown here (with individual cells) should apply to modules.

The measured current gains illustrate the potential of using nanoPAN reflectors to enhance the ground albedo and increase the efficiency of fielded bifacial PVs. Simulations indicate that increasing the albedo by 0.5, consistent with this work, is expected to improve the power output by 18 % under standard conditions [6] and the electricity production (annual energy yield) by 30–40 % (global average) [4].

## 4 Conclusions

We have fabricated and experimentally investigated the performance of an artifical ground reflector capable of daytime radiative cooling. An electrospun layer of beaded nanofibers (nanoPAN) optimizes solar-specific scattering, resulting in a ∼20 W/m^2^ gain in daytime radiative cooling power compared a PDMS-coated silver mirror. Additionally, the nanoPAN-based reflector increases the ground albedo compared to sand, resulting in 6.42 mA/cm^2^ of additional photocurrent generated by a commercial silicon solar cell. Future work involves investigating how the radiative-cooling ground reflector impacts the power output and temperature of fielded bifacial solar panels at the appropriate scale to capture the effects of higher albedo and lower ground-surface temperatures on the microclimate at the PV site.

## Supplementary Material

Supplementary Material Details
