# Supplementary material for: High albedo daytime radiative cooling for enhanced bifacial PV performance
Source: Nanophotonics. 2023 Dec 14;13(5):621–7. doi: 10.1515/nanoph-2023-0611 (PMC11501292; doi:10.1515/nanoph-2023-0611)
Supplement: Supplementary file 1 — Supplementary Material Details [file j_nanoph-2023-0611_suppl_001.pdf]

Supplementary Information for High Albedo Daytime Radiative Cooling for Enhanced Bifacial PV Performance

1. DIFFUSE REFLECTANCE OF NANOPAN REFLECTOR

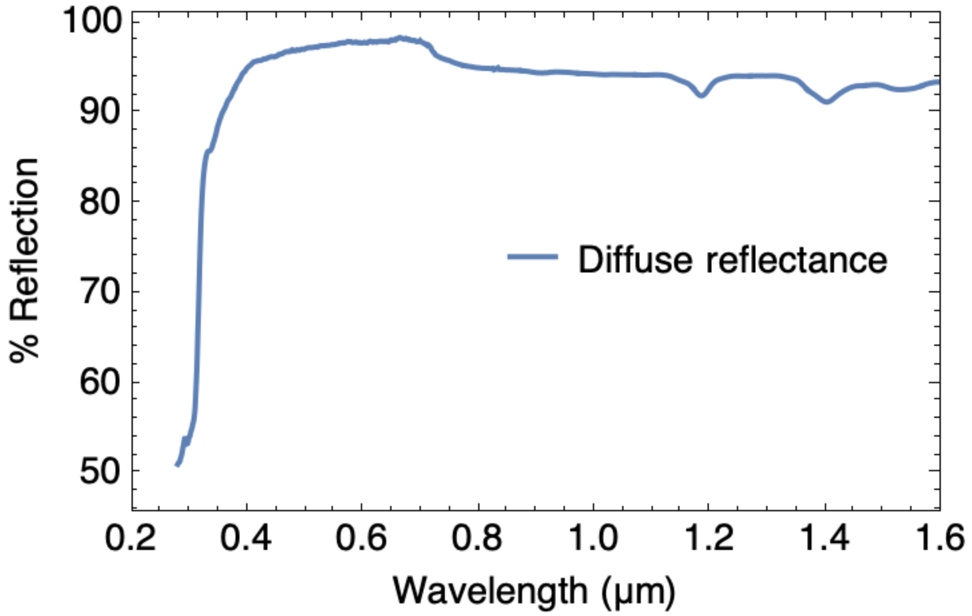

Fig. S1. Diffuse reflectance of the nanoPAN-based reflector (nanoPAN/PDMS/Ag) determined using UV-Vis measurements equipped with an integrating sphere (Shimadzu, UV-3600Plus).

2. NANOPAN COATING ON ALUMINUM SHEETS

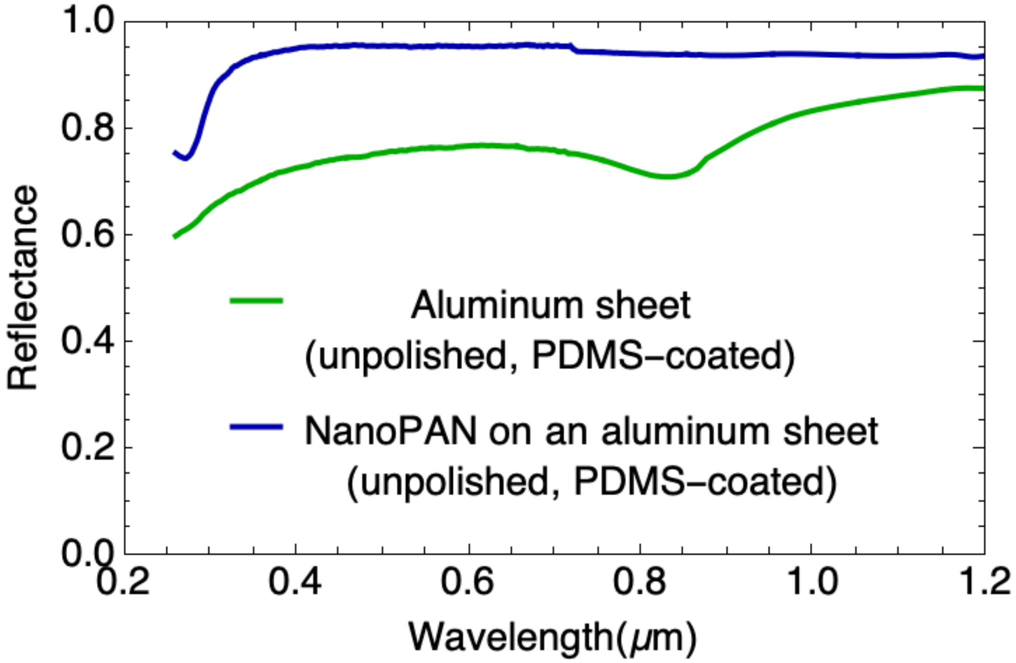

Fig. S2. Applying the nanoPAN layer to an aluminum sheet significantly enhances the total solar reflectance.
